# Reproducibility and clinical significance of pre-ovulatory serum progesterone level and progesterone/estradiol ratio on the day of human chorionic gonadotropin administration in infertile women undergoing repeated in vitro fertilization cycles

**DOI:** 10.1186/s12958-015-0037-9

**Published:** 2015-05-13

**Authors:** Yu-Ju Lin, Kuo-Chung Lan, Fu-Jen Huang, Pin-Yao Lin, Hsin-Ju Chiang, Fu-Tsai Kung

**Affiliations:** Department of Obstetrics and Gynecology, Kaohsiung Chang Gung Memorial Hospital and Chang Gung University College of Medicine, Kaohsiung, Taiwan; Department of Obstetrics and Gynecology, Chang Gung Memorial Hospital, Chia Yi, Taiwan

**Keywords:** Pre-ovulatory serum progesterone, Progesterone/estradiol ratio, In vitro fertilization, Reproducibility

## Abstract

**Background:**

The objective of this study was to explore the cycle-to-cycle reproducibility of serum progesterone level and progesterone/estradiol (P/E2) ratio in the final step of triggering oocyte maturation in patients undergoing repeated consecutive controlled ovarian hyperstimulation for in vitro fertilization (COH-IVF) treatment and to investigate the clinical parameters associated with serum progesterone concentration and P/E2 ratio.

**Methods:**

We retrospectively studied 524 cycles in 203 infertile women who underwent two or more fresh COH-IVF cycles from July 1998 to May 2012 in a university hospital IVF unit. The patients were divided into groups according to the number (2, 3 or > = 4) of total successive IVF cycles with successful oocyte retrieval. The within-subject reproducibility of serum P and P/E2 was tested by calculating intra-class correlation coefficients (ICCs). Multiple linear regression analysis was used to assess the association between patient variables and pre-ovulatory serum P level and P/E2 ratio.

**Results:**

The ICCs in women who underwent 2, 3 and > = 4 IVF cycles were −0.052, 0.163 and 0.212, respectively, for serum P concentration and 0.180, 0.168 and 0.148, respectively, for P/E2 ratio. All ICCs for both serum P and P/E2 ratio were indicative of poor reproducibility. The number of oocytes was significantly positively related to P concentration, and endometrial thickness was significantly negatively related to P concentration and P/E2 ratio.

**Conclusion:**

The cycle-to-cycle reproducibility of pre-ovulatory serum P concentration and P/E2 ratio was poor in individual patients, and these fluctuations were more cycle- than patient-dependent. The number of oocytes was the most significant factor relating to P concentration. By using milder stimulation approach to produce fewer oocytes in the next cycle is a strategy to overcome the high serum P concentration, while clinicians should consider each patient’s general condition including the age, ovarian reserve, embryo grading and the capacity of frozen-thawed embryo transfer.

## Background

It is well known that gonadotropin-releasing hormone (GnRH) agonists and antagonists, known as GnRH analogues, can effectively eradicate the premature luteinizing hormone surge, and they are almost routinely used during controlled ovarian hyperstimulation (COH) in modern in vitro fertilization (IVF) treatment. However, unpredicted elevation of serum progesterone without an increase luteinizing hormone (LH) in the late follicular phase still occurs in patients co-treated with GnRH-a and COH, a phenomenon called premature progesterone rise (PPR) [1]. The reported incidence of PPR varies greatly from 3 % to 71 %, and it has been reported to be as high as 35 % in GnRH agonist cycles and 38 % in antagonist cycles [2–5]. PPR has previously been defined based on absolute progesterone concentration or as a progesterone/estradiol (P/E_2_) ratio on the day of human chorionic gonadotropin (hCG) administration [6–8]. The effects of elevated serum progesterone on oocyte maturation, endometrial development and receptivity, and prognostic outcomes after embryo transfer are controversial. An increasing number of retrospective studies suggest that progesterone elevation on the day of hCG administration may have a negative effect on pregnancy rate, and this hypothesis was confirmed by a meta-analysis based on the analysis of 60,000 IVF cycles [9–11]. However, PPR remains a controversial issue because several studies did not find this association [6, 12, 13]. In addition, whether the level of serum progesterone in consecutive cycles remains constant or fluctuates in an individual undergoing repeated COH-IVF treatment is unknown.

In this study, we aimed to explore the inter-cycle reproducibility of serum progesterone and P/E_2_ ratio in the final step of triggering oocyte maturation in patients undergoing repeated consecutive COH-IVF treatment. The association of both serum progesterone concentration and P/E_2_ ratio, and the patient’s clinical characteristics and ovarian stimulation responses were also assessed.

## Methods

### Subjects

This retrospective, single-center cohort study involved consecutive infertile couples who underwent two or more fresh IVF and/or intracytoplasmic sperm injection-embryo transfer (ICSI-ET) cycles from July 1998 to May 2012 in our IVF unit. Women older than 45 years of age and those with intervals of more than 5 years from the first to the last IVF cycle were excluded. The patients were divided into groups according to the number (2, 3 or ≥ 4) of total successive IVF cycles with successful oocyte retrieval. The patients’ characteristics included age, body mass index, basal follicle-stimulating hormone (FSH), luteinizing hormone, estradiol levels on cycle day 3, and causes of infertility. The study was approved by the Institutional Review Board of the Ethics Committee of Chang Gung Medical Foundation, Taiwan in March 2014 (CGMF IRB No.: 103-1156B).

### Ovarian stimulation protocols

All women received a GnRH-a long protocol, a GnRH-a short protocol or a GnRH antagonist protocol depending on ovarian reserve, which was assessed by the patient’s age, baseline serum FSH concentration, previous ovarian response to gonadotropins, and the preference of each clinician. The initial dose of gonadotropin was individualized (varied from 75 to 450 IU) for each patient using either human menopausal gonadotropin (hMG) or FSH (purified or recombinant), with further dose adjustments based on each patient’s ovarian response as assessed by serum estradiol concentration and sonographic monitoring of follicular growth. In the GnRH antagonist protocol, 0.25 mg of cetrorelix acetate (Cetrotide®; Serono, Baxter Oncology GmbH, Halle, Germany) was administered daily starting on day 6 of ovarian stimulation or when the lead follicle reached ≥ 14 mm in diameter; administration continued until the day of hCG injection. When the lead follicle reached 16–18 mm in diameter, hMG and FSH were discontinued, and hCG (Ovidrel®; Serono, Modugno, Italy) was administered. Oocyte retrieval was scheduled 36–38 h later. Standard IVF or ICSI procedures, depending on semen parameters, were used to achieve oocyte fertilization, as previously described [14, 15].

On day 3, all embryos were graded on a scale of 0 to 4, which was based on a modification of Veeck's morphological grading system and our previous report [16, 17]. Embryo transfer was performed 3 to 5 days after oocyte retrieval, and no more than 4 embryos were transferred per cycle. During the luteal phase, each patient received 800 mg/day of micronized progesterone (Utrogestan; Piette International Laboratories, Brussels, Belgium) intravaginally or 90 mg of progesterone vaginal gel once daily (Crinone 8 %; Serono Pharmaceuticals Ltd., UK) starting on the day after oocyte retrieval. Clinical pregnancy was defined as one or more gestational sacs detected by transvaginal ultrasound.

### Hormone measurements

Serum P and E_2_ were measured on the day of hCG administration of each IVF cycle using a commercially available immunoassay system (ADVIA Centaur ® XP, Siemens, USA). The lower limits of detection were 0.15 ng/mL for P and 11.8 pg/mL for E_2_. The intra- and inter-assay coefficients of variation were 5.2 % and 3.5 %, respectively, for P and 5.0 % and 4.1 %, respectively, for E_2_. The P/E_2_ ratio was calculated as P (ng/mL) x 1,000/E_2_ (pg/mL).

### Statistical analysis

All statistical analyses were performed using SPSS (ver. 17.0; Statistical Package for Social Sciences, Inc., Chicago, IL, USA). Continuous data are reported as the mean ± standard deviation and compared in the three groups by one—way analysis of variance (ANOVA), whereas categorical variables were compared by the *χ*^2^ or Fisher’s exact tests. Fisher’s least significant difference post hoc test was applied when ANOVA revealed statistical significance. To assess the central tendency and distribution of the measurements, we visualized pre-ovulatory serum P concentrations and P/E_2_ ratios per cycle within the same patient using box plots. The within—subject reproducibility of serum P and P/E_2_ was estimated by calculating intra-class correlation coefficients (ICCs) and their 95 % confidence intervals [18]. ICCs were defined as the ratio of between-subject variability to total variability, the latter including between—and within-subject variability. ICC values ≥ 0.75, between 0.60 and 0.74, between 0.40 and 0.59 and < 0.40 or a negative value were interpreted as excellent, good, fair, and poor, respectively [19, 20]. Multiple linear regression analysis was used to assess the association between patient variables and pre-ovulatory serum P level or P/E_2_ ratio. All *P* values were two-sided, and a *P* value of less than 0.05 was considered to be statistically significant.

## Results

A total of 524 fresh IVF cycles in 203 infertile women were included in the analysis, and the mean interval from the first to the last IVF cycle was 19.1 ± 15.5 months. Of these 203 women, 125 (61.5 %) underwent two fresh IVF cycles, 52 (25.6 %) underwent three cycles, and 26 (12.8 %) underwent four to seven IVF cycles. The patient baseline demographic, cycle characteristics and cycle outcomes of the three groups are summarized in Table 1.

**Table 1:** Patient baseline demographics, cycle characteristics and cycle outcomes of the three groups according to the number of total in vitro fertilization cycles

Variable	Overall	2 cycles	3 cycles	≥ 4 cycles	*P value*
Baseline demographics					
No. of cases (cycles)	203 (524 cycles)	125 (250 cycles)	52 (156 cycles)	26 (118 cycles)	
Age (years)	34.3 ± 4.7	33.7 ± 4.4 ^a^	33.7 ± 4.9 ^b^	36.3 ± 4.4 ^ab^	< 0.001
Mean interval from 1st to last IVF cycle (months)	19.1 ± 15.5	14.2 ± 14.2 ^ab^	24.7 ± 13.5 ^a^	31.3 ± 15.0 ^b^	< 0.001
BMI (kg/m^2^)	21.5 ± 2.8	21.7 ± 3.1	21.2 ± 2.3	21.5 ± 2.7	0.220
Infertility factors					0.003
Male factor	194 (34 %)	86 (33 %)	58 (35 %)	50 (38 %)	
Tubal factor	119 (21 %)	59 (22 %)	42 (25 %)	18 (14 %)	
Ovulatory factor	75 (13 %)	37 (14 %)	15 (9 %)	23 (18 %)	
Endometriosis factor	87 (15 %)	32 (12 %)	37 (22 %)	18 (14 %)	
Unexplained factor	89 (16 %)	51 (19 %)	16 (9 %)	22 (17 %)	
Cycle characteristics					
IVF protocol					< 0.001
GnRHa long protocol	351 (67 %)	179 (72 %)	116 (74 %)	56 (48 %)	
GnRHa short protocol	130 (25 %)	48 (19 %)	31 (20 %)	51 (43 %)	
Antagonist protocol	38 (7 %)	22 (9 %)	6 (4 %)	10 (9 %)	
ART method (n)					0.132
Conventional IVF	336 (64 %)	171 (68 %)	92 (59 %)	73 (62 %)	
ICSI	188 (36 %)	79 (32 %)	64 (41 %)	45 (38 %)	
Duration of stimulation (days)	9.3 ± 1.8	9.4 ± 1.8	9.1 ± 1.7	9.4 ± 2.0	0.379
No. of gonadotrophin ampoules used	34.6 ± 14.5	32.5 ± 11.8 ^a^	33.3 ± 13.7 ^b^	40.8 ± 18.8 ^ab^	< 0.001
Estradiol (pg/mL) on hCG day	2177.3 ± 1441	2377.4 ± 1541 ^a^	2360.5 ± 1443 ^b^	1505.4 ± 938 ^ab^	< 0.001
No. of follicles ≥ 16 mm on hCG day	4.91 ± 3.1	5.25 ± 3.1 ^a^	5.23 ± 3.4 ^b^	3.77 ± 2.4 ^ab^	< 0.001
Number of oocytes retrieved	6.9 ± 3.9	7.7 ± 4.0 ^a^	7.2 ± 3.9 ^b^	5.0 ± 3.1 ^ab^	< 0.001
EM (mm) on hCG day	13.0 ± 3.1	13.0 ± 3.0 ^a^	13.5 ± 3.5 ^b^	12.3 ± 2.5 ^ab^	0.011
Pre-ovulation serum progesterone (ng/mL)	1.62 ± 0.6	1.72 ± 0.6 ^a^	1.62 ± 0.7 ^b^	1.41 ± 0.6 ^ab^	< 0.001
P/E_2_ ratio	1.11 ± 1.2	1.13 ± 1.3	1.02 ± 1.1	1.19 ± 0.7	0.557
Cycle outcomes					
Normal fertilization rate (%)	77.4 (2830/3655)	77.1 (1488/1928)	76.2 (865/1134)	80.4 (477/593)	0.778
Mean no. of embryos transferred	2.5 ± 1.0	2.5 ± 1.0	2.5 ± 1.1	2.3 ± 1.1	0.106
Mean embryo score per embryo (Day 3)	3.3 ± 1.0	3.4 ± 0.9 ^a^	3.3 ± 0.9 ^b^	3.0 ± 1.2 ^ab^	0.001
Implantation rate (%)	15.8 (208/1316)	18.7 (120/642) ^a^	16.0 (64/399) ^b^	8.7 (24/275) ^ab^	0.004
Clinical pregnancy rate / transfer cycle (%)	33.7 (164/486)	40.0 (94/235) ^a^	34.2 (50/146) ^b^	19.0 (20/105) ^ab^	0.022
Live birth rate / transfer cycle (%)	19.3 (94/486)	25.1 (59/235) ^a^	18.4 (27/146) ^b^	7.6 (8/105) ^ab^	0.006

*Note*: Values are mean ± SD or proportion. Figures sharing the same superscript (a, b) are statistically significantly different at P < 0.05 by LSD analysis post hoc

No, number; IVF, in vitro fertilization; BMI, body mass index; ART, assisted reproductive technology; ICSI, intracytoplasmic sperm injection; hCG, human chorionic gonadotropin; EM, endometrial thickness; P/E_2_, progesterone/estradiol

The mean serum P concentration and P/E_2_ ratio on the day of hCG administration were 1.62 ng/mL and 1.11, respectively. As expected, the women who had more IVF treatment cycles tended to be older and had longer intervals between IVF treatments, particularly for patients who underwent ≥ 4 IVF cycles. In addition, the main IVF protocol also changed, from the GnRHa long protocol to the short and antagonist protocols. There were no differences among the three groups in body mass index, method of assisted reproductive technology (ART), duration of ovarian stimulation and normal fertilization rate. Interestingly, the pre-ovulatory serum P concentration was significantly lower among patients who underwent more IVF cycles, while the P/E_2_ ratio was similar among the three groups. Peak E_2_ concentrations, number of follicles ≥ 16 mm in diameter, and the number of oocytes retrieved were higher among women who underwent 2 or 3 IVF cycles than among those who underwent ≥ 4 cycles. In contrast, women who underwent ≥ 4 IVF cycles required a higher dose of gonadotropin but had fewer follicles than women who underwent 2 or 3 IVF cycles. In addition, fewer oocytes were collected, the endometrium was thinner, and the mean embryo score per embryo was lower in women who underwent ≥ 4 IVF cycles. These women also had lower rates of implantation, clinical pregnancy and live birth despite an equal number of embryos being transferred.

**Fig. 1:**
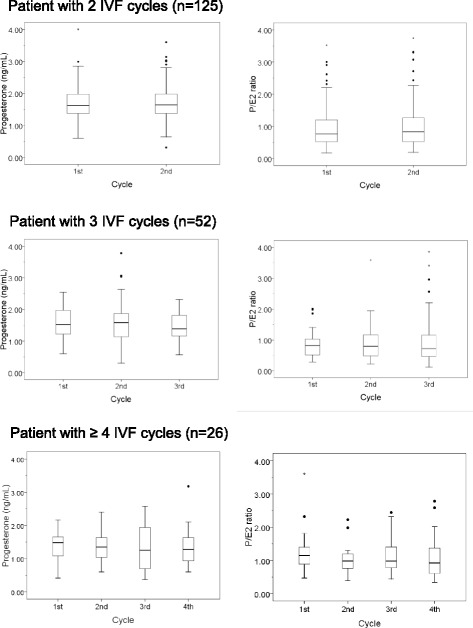
Box and whiskers plots depicting distribution of pre-ovulatory serum progesterone concentration and P/E_2_ ratio on the day of hCG administration across 2 ~ 4 IVF cycles in each group of patients

The distributions of serum P concentration and P/E_2_ ratio on the day of hCG administration across 2 ~ 4 consecutive IVF cycles are depicted in Fig. 1. The median serum P concentrations in the 125 women who underwent 2 IVF cycles each were 1.61 ng/mL in the first cycle and 1.64 ng/mL in the second cycle, and the median P/E_2_ ratios were 0.76 and 0.83, respectively. In the 52 patients who underwent 3 IVF cycles, the median serum P concentration ranged from 1.39 to 1.58 ng/mL, and the median P/E_2_ ratio ranged from 0.70 to 0.80, with both showing similar distribution patterns in each cycle. However, the box plots of the patients who underwent 4 IVF cycles showed markedly different distributions across individual cycles.

The ICC data are summarized in Table 2. The ICCs among women who underwent 2, 3, and ≥ 4 IVF cycles were −0.052, 0.163 and 0.212, respectively, for serum P concentration and 0.180, 0.168 and 0.148, respectively, for P/E_2_ ratio. All of these ICCs were indicative of poor reproducibility.

**Table 2:** Intraclass correlation coefficients of pre-ovulatory serum progesterone concentration and P/E_2_ ratio in patients who underwent repeated consecutive in vitro fertilization cycles

	Intraclass correlation coefficient (ICC)		
	2 cycles	3 cycles	≥ 4 cycles
Number of patients (cycles)	125 (250 cycles)	52 (156 cycles)	26 (118 cycles)
Pre-ovulatory serum P on hCG day (95 % CI)	−0.052 (−0.24 ~ 0.14)	0.163 (−0.01 ~ 0.37)	0.212 (0.00 ~ 0.52)
P/E_2_ ratio on hCG day (95 % CI)	0.180 (−0.01 ~ 0.36)	0.168 (−0.13 ~ 3.77)	0.148 (−0.06 ~ 0.46)

CI, confidence interval

Multiple linear regression analysis showed that after adjusting for possible confounders, serum P concentration was positively correlated with E_2_ concentration (*P* = 0.009) and the number of oocytes retrieved (*P* < 0.001) and negatively correlated with endometrial thickness (*P* = 0.043) (Table 3). Additionally, after adjusting for possible confounders, the P/E_2_ ratio was positively correlated with duration of stimulation (*P* < 0.001) and negatively correlated with the number of oocytes retrieved (*P* = 0.002) and endometrial thickness (*P* = 0.012) (Table 4).

**Table 3:** Summary of multiple linear regression analysis for parameters associated with pre-ovulatory serum progesterone

Parameter	B	SE (B)	β	Sig.(*p*)
Age (years)	−0.010	0.007	−0.022	0.677
Body mass index (kg/m^2^)	0.000	0.011	−0.003	0.955
Number of gonadotropin ampoules used	0.003	0.003	0.076	0.234
Duration of stimulation (day)	0.025	0.021	0.069	0.244
Estradiol (pg/mL) on hCG day	6.995E-5	0.000	0.152	0.009
Number of oocytes retrieved	0.039	0.010	0.235	< 0.001
Endometrium thickness (mm)	−0.021	0.010	−0.098	0.043
R^2^	0.111			
F	7.208*			

* *P* < 0.001

**Table 4:** Summary of multiple linear regression analyses for parameters associated with P/E_2_ ratio

Parameter	B	SE (B)	β	Sig.(*p*)
Age (years)	0.003	0.014	0.012	0.818
Body mass index (kg/m^2^)	0.023	0.020	0.055	0.256
Number of gonadotrophin ampoules used	−0.009	0.005	−0.106	0.099
Duration of stimulation (day)	0.156	0.039	0.238	< 0.001
Number of oocytes retrieved	−0.051	0.016	−0.166	0.002
Endometrium thickness (mm)	−0.049	0.019	−0.123	0.012
R^2^	0.086			
F	6.396*			

* *P* < 0.001

## Discussion

To the best of our knowledge, this study is the first to assess the inter-cycle reproducibility of pre-ovulatory serum P measurements and P/E_2_ ratio in individual women who underwent multiple IVF/ICSI cycles. Our major finding is that the cycle-to-cycle reproducibility of both parameters was poor in individual patients. These inter-cycle variations during repeated IVF cycles suggest that neither serum P concentration nor P/E_2_ ratio in a subsequent IVF cycle can be predicted from the results in a previous cycle. Factors significantly associated with serum P concentration included E_2_ concentration on the day of hCG administration, the number of oocytes collected and the thickness of the endometrium; factors significantly associated with the P/E_2_ ratio included the duration of stimulation, the number of oocytes retrieved and the thickness of the endometrium.

The possible mechanism of PPR is likely due to an excess number of mature follicles, each of which produces a normal amount of P during the late follicular phase of COH [9, 12, 21]. We found that pre-ovulatory serum P concentrations were significantly higher among women who underwent 2 or 3 cycles than among those who underwent ≥ 4 IVF cycles. In addition, the peak E_2_ concentration was higher and more oocytes were collected in those who underwent 2 or 3 cycles than in those who underwent ≥ 4 IVF cycles. Multiple regression analysis showed that serum P concentration was positively correlated with both the number of oocytes retrieved and the peak E_2_ level, which is consistent with the results of previous studies and with the above hypothesis [9, 11]. Thus, it is an option to overcome PPR by using milder stimulation approach to produce fewer oocytes in the next cycle. However, clinicians should consider each patient’s general condition including the age, ovarian reserve, embryo grading and the capacity of frozen-thawed embryo transfer.

We also observed a negative correlation between serum P concentration and endometrial thickness, which may indicate advanced endometrial maturation. Advanced maturation of the endometrium, which has been reported during the peri—and post-ovulatory periods of the stimulation cycle [22–24], may be due to several factors, including supra-normal estradiol concentration, early and increased exposure of the endometrium to P and to hCG injections [25–28]. Other studies have implied that endometrial thickness and pattern may be useful indicators of endometrial receptivity, and they have shown that a premature secretory endometrial pattern is introduced by elevation of P. Advanced endometrial maturation may be unfavorable for implantation and have an adverse effect on pregnancy rates [29–32].

In this study, we retrospective analysis of a subgroup of patients who had undergone repeated IVF/ICSI cycles measured pre-ovulatory serum P concentration and P/E_2_ ratio over multiple IVF cycles in individual patients. Women who had undergone ≥ 4 IVF cycles showed relatively poor responder trend, which may be related to aging. Older women have poorer ovarian responses and yield fewer good quality embryos and fewer available embryos to be frozen than younger women. Increasing the number of attempted IVF cycles can result in a higher cumulative pregnancy rate. However, while IVF may largely overcome infertility in younger women, it does not reverse the age-dependent decline in fertility, especially in women aged ≥ 40 years [33]. Our findings suggest that a maximum of 4 treatment cycles may present a critical point in IVF because of the extremely low ovarian response and poor pregnancy outcomes occurring subsequently.

The relationship between PPR and pregnancy outcome remains the subject of much debate, and some studies have hypothesized that the possible detrimental effect of PPR is mainly through a negative impact on endometrial development at the end of the follicular phase rather than by negatively affecting embryo quality. This may result in embryo-endometrial asynchrony with poor endometrial receptivity [10, 34, 35], and this may reduce embryo implantation and pregnancy rates [24, 25]. Thus, freezing embryos and transferring them in a subsequent frozen-thawed cycle may avoid impairments in endometrial receptivity, which is why it is the method most frequently used to manage PPR.

The results of an oocyte donation program yielded similar observations as the present study. In that study, among 120 women who each donated oocytes on two occasions, the serum P level was ≥ 1.2 ng/mL in the first donation cycle but < 1.2 ng/mL (no further serum P elevation) in the second cycle. The study also reported that PPR did not have a negative impact on pregnancy rate [34]. Notably, these results provide additional evidence supporting inter-cycle variations in pre-ovulatory serum P concentration among women undergoing repeated oocyte donation IVF cycles.

No general consensus has been reached on the cut-off value for PPR. Studies designed to determine the most appropriate cut-off value have utilized receiver operating characteristic (ROC) analysis, trend analysis or arbitrary methods. In addition to pre-ovulatory serum P concentration, some studies have used P/E_2_ ratio to diagnose PPR, however, the optimal cut-off value for P/E_2_ ratio has been reported to range from 0.55 to 1.2 and P/E_2_ ratio has not been shown to definitively predict IVF outcomes [6–8, 36]. We have previously presented data suggesting that progesterone concentration is not the only factor determining the clinical pregnancy and live birth rates, and we have found that PPR has an obvious negative impact on pregnancy outcome only at extremely high progesterone concentration (P ≥ 1.94 ng/mL) [37].

There were considerable inter-cycle variations in serum P concentration and P/E_2_ ratio on the day of hCG administration in the current study; the changes were bidirectional and went either down or up. Further questions, such as whether women who always have constant “normal” pre-ovulatory serum P levels in repeated IVF cycles have better pregnancy outcomes, remain unresolved. Our study did not assess the recurrence of PPR. The design of the study had several potential biases, such as three different stimulation protocols used and different types of gonadotropin preparations for ovarian stimulation, which may mask the possible effect on serum P concentration and the cycle outcome. Another limitation is the small number of patients undergoing repeated IVF cycles. Further studies are required to answer the unsolved questions mentioned above, investigate the effects of recurrent PPR, and re-examine the reproducibility and potential clinical meaning of these parameters.

## Conclusions

Cycle-to-cycle reproducibility of pre-ovulatory serum P concentrations and P/E_2_ ratio was poor in individual patients, and the fluctuations were more cycle- than patient-dependent. The number of oocytes was the most significant factor relating to the P concentration. By using milder stimulation approach to produce fewer oocytes in the next cycle is a strategy to overcome PPR, while clinicians should consider each patient’s general condition including the age, ovarian reserve, embryo grading and the capacity of frozen-thawed embryo transfer.

## Abbreviations

ART: Assisted reproductive technology
COH: Controlled ovarian hyperstimulation
ET: Embryo transfer
FSH: Follicle-stimulating hormone
GnRHa: Gonadotropin-releasing hormone agonist
hCG: human chorionic gonadotropin
hMG: human menopausal gonadotrophins
ICCs: Intra-class correlation coefficients
IVF: In vitro fertilization
ICSI: Intracytoplasmic sperm injection
LH: luteinizing hormone
P: Progesterone
P/E_2_: Progesterone/estradiol
PPR: Premature progesterone rise
